# The Challenges of Life Design Counseling in the Times of the Coronavirus Pandemic (COVID-19)

**DOI:** 10.3389/fpsyg.2020.01235

**Published:** 2020-07-14

**Authors:** Ya Wen, Huaruo Chen, Kai Li, Xueying Gu

**Affiliations:** ^1^School of Education Science, Nanjing Normal University, Nanjing, China; ^2^Center for Research and Reform in Education, Johns Hopkins University, Baltimore, MA, United States; ^3^School of Psychology, Nanjing Normal University, Nanjing, China

**Keywords:** life design counseling, self-construction theory, career construction theory, challenges, counselors

## Introduction

In the face of globalization and the rapid development of information technology, individuals are in a world in which Volatility, Uncertainty, Complexity, and Ambiguity (VUCA) reign (Millar et al., 2018), and employment has become increasingly unstable and uncertain. Especially COVID-19 has become a global pandemic, leading to economic recession and massive unemployment. It is important for everyone to design their own lives. Based on this situation, the previous guidance of personal career choice has become more difficult to meet people's needs, and individuals need to improve their career adaptability and manage their careers, especially in designing own lives (Rudolph et al., 2017). Life Design Counseling (LDC) aims to help clients build a personally meaningful life and has the following advantages: firstly, it provides a new perspective for career counseling; secondly, it exerts significant indication on cross-cultural research; finally, it emphasizes the preventive function of career counseling. LDC faces three most important challenges: many people who need help do not have access to targeted LDC; lack of specifically trained LDC counselors; and lack of suitable LDC assessment methods. The unique contribution of this paper is as follows: (1) put forward the challenges faced by LDC in a clear and detailed way and (2) add some targeted countermeasures that can be tried to take in the face of challenges.

## Life Design Counseling

### What Is Life Design Counseling?

LDC is a career intervention that helps the clients create a life story and discover an understanding of a life theme so that the clients can rewrite individuals' narrative identity, clarify individuals' self-concept, establish a life purpose, and explore a path of action (Savickas et al., 2009; Savickas, 2011a,b). The theoretical basis of LDC is Guichard's self-construction theory and Savikcas' career construction theory. Both theories involve social constructivism, but there are some differences between the two theories as follows (Savickas, 2012; Guichard, 2016a): the former focuses on the survival direction of the individual, emphasizes the individual's dynamic development based on System of Subjective Identity Forms (SSIF), and constructs self-concepts; the latter points out that career can be a dynamic construction process, and subjective self and external world can adapt to each other, counselors assist the clients to reconstruct life meaning and identity, and discover the theme of life, so individuals need to solve three key questions about what kind of career path to build, how to build a career path, and why to build a career path (Savickas, 2008; Guichard, 2009; Savickas et al., 2018). LDC based on career construction theory is more suitable for mature adults; LDC based on the self-construction theory is more suitable for teenagers or emerging adults (Di Fabio, 2010; Guichard, 2016b). According to these, everyone needs to design their life, so providing LDC is feasible, operable, and necessary.

Savickas et al. (2009) proposed that the intervention model of life design includes six steps: the first step is to determine the problem and counseling objectives by clients and counselors; the second step is for the clients to explore current subjective identity; the third step is to translate clients' narrative into clear content; the fourth step is to put the question into a new story; the fifth step is to ask the clients to develop an identity-related action plan; and the final step is short and long-term follow-up (Maree, 2015a,b). Based on LDC, counselors help clients formulate career problems, explore role models, etc., then construct themes, reconstruct micro-narratives into macro-narratives, and rebuild narrative identity with greater coherence and continuity (Savickas, 2015; Taylor and Savickas, 2016). Currently, LDC is mainly based on Savikcas' career construction interview (CCI) and Guichard's life and career design dialogues (LCDD). CCI, for example, lists several core questions: role models for growing up; favorite magazine or TV program, etc. (Barclay and Wolff, 2012; Barclay et al., 2019).

### Advantages of Life Design Counseling

#### Provide a New Perspective of Career Counseling

LDC provides a new perspective on career counseling for clients in this fast and changing world (Jacquin and Juhel, 2017; Jiang et al., 2019). In the past, career counseling has focused on the professional needs of individuals of certain age or role. However, nowadays, LDC not only focuses on the career needs of different ages from adolescent to adult but also of different roles, such as employees and family members, emphasizing career from a lifelong, holistic, and dynamic perspective.

#### Apply to Cross-Cultural Researches

In the past, many counseling methods had difficulties in applying to cross-cultural researches. However, LDC has conducted a series of cross-cultural researches successfully in the United States, France, Italy, Portugal, Turkey, China, and other countries (Briddick et al., 2018; Tian et al., 2020).

#### Play the Preventive Role of Career Counseling

Previous career counseling often serviced individuals faced with career dilemma, such as those people swaying in the career transition and those with low career self-efficacy (Bikos et al., 2013). LDC attempts to help individuals discover the life theme from the narrative process and build and create careers in the long run, not just focus on the current career challenges.

## Challenges in Life Design Counseling

### Many People in Need Cannot Get Targeted LDC

Many people cannot get targeted LDC. In fact, individuals engaged in different occupations or at different stages, and everyone needs LDC, whether adolescents, college students, workers, vulnerable groups, etc. (Maree, 2014, 2016a; Maree and Twigge, 2016). As a new form of counseling, LDC plays an important role to discover life themes and establish subjective identity.

If individuals or groups cannot get targeted LDC, they will encounter many difficulties, such as lack of vocational certainty, difficulty in re-employment after unemployment, and reduced quality of life (Maree et al., 2018a; Cardoso and Sales, 2019). On the contrary, individuals would benefit greatly if targeted LDC was made available to those in need. Through LDC, college students' career uncertainty and career indecision are reduced; adolescents' career self-efficacy and life satisfaction are significantly improved; and vulnerable people can improve their quality of life (Barclay and Stoltz, 2016a; Setlhare and Wood, 2016; Cardoso et al., 2017). Special attention should be paid to the fact that career adaptability has been recorded as a key self-regulation process in career construction and life design in the twenty-first century. However, some studies found that after LDC intervention, career adaptability has not changed (Savickas and Porfeli, 2012; Maree et al., 2018b; Maree, 2019). Only a few studies have found that LDC can improve career adaptability or some dimensions (Nota et al., 2016; DaSilva et al., 2020).

However, due to the fact that groups of different regions, ages, and genders have different career needs, it is still difficult to provide targeted LDC for specific individuals or groups. An LDC study of Italian entrepreneurs found that only such a small number of samples may misrepresent the whole country or other types of entrepreneurs (Di Fabio et al., 2012). Other scholars point out that people from different stages of career development are needed for future research (Cardoso et al., 2016a,b). For individual counseling, counselors need to give targeted counseling based on personal needs; for group counseling, it is better if the clients have similar needs, which may lead to better group motivation. To sum up, many people in need cannot get targeted LDC, which is one of the key challenges.

### Lack of Specifically Trained LDC Counselors

There is a lack of specifically trained LDC counselors in the current society. In a case study, the counselors are recognized experts in the field of LDC, who have many years of theoretical and practical experience (Cardoso et al., 2014). In another study, the counselors are psychologists with more than 30 years of experience in career counseling (Maree, 2020a). A counselor in a study on college students is studying for a Ph.D. degree in psychology (DaSilva et al., 2020). Counselors in the existing research generally have a masters or doctorate degree and have rich counseling experience, so such professional LDC counselors are still rare.

Some researchers have found that if LDC counselors are not professional and especially lack active listening skills and empathy to clients, it would become difficult for counselors to be sensitive to comprehend problems of clients and take active roles in the development of reflexivity of clients (Guichard et al., 2012). Highly specialized LDC counselors could contribute to the client's innovative moments (IMs) and are more likely to use metaphors to identify the emotional needs of clients (Savickas, 2016; Savickas and Pouyaud, 2016), which play an important role in the change of clients (Cardoso et al., 2019, 2020).

In conclusion, how to establish a standardized competency framework for LDC counselors and train a certain number of professional life design counselors is also an important challenge.

### Lack of Assessment Methods for LDC

As a form of counseling, the process and effect evaluation of LDC are very important, which related to the current career status and the subsequent career construction of the client (Savickas and Guichard, 2016). If there is no suitable evaluation procedure, the impact of LDC will be unknown; if there is an assessment of LDC, researchers will have a better understanding of how LDC affects individuals or groups (Barclay and Stoltz, 2016b).

However, there are still some difficulties in designing an LDC assessment plan. Clients are unique which made it become more difficult to use multiple variables to evaluate changes in clients before and after counseling. In traditional research, the experimental group was compared with the control group, and the experimental group was statistically significant, proving that the treatment was effective. This kind of empirical design is not suitable for career counseling, which is important in referring to methods in mathematics, economics, and biology, trying to evaluate LDC in a simulated fractal model rather than relying on any single-outcome variable in the counseling assessment (Savickas et al., 2009).

In addition, some researchers have used qualitative assessments and found that estimating is not easy. Lengelle et al. (2016) introduced LDC through creative writing by Interpersonal Process Recall (IPR) but did not elaborate on how to evaluate the effect. Di Fabio (2016) used the qualitative research tool Career Counseling Innovative Outcomes (CCIO) coding system to describe the changes of clients through LDC; results showed that the subjective understanding of researchers affected the objectivity of counseling. Cardoso et al. (2017) conducted LDC on adolescents and found that future studies need to combine qualitative evaluation with quantitative evaluation. Some authors recommended an integrative qualitative + quantitative approach to LDC by combined Career Interest Profile (CIP) and Maree Career Matrix together (Maree, 2016b, 2020b; Maree and Taylor, 2016). However, those kinds of assessments also face challenges, such as a small sample size and lack of longitudinal research. Nota et al. (2016) indicated that researches should include follow-up studies, such as follow-up at 6 months. In summary, lack of a suitable evaluation of LDC is a major challenge.

## Discussion

LDC has played an important role in career counseling. However, it cannot be neglected that LDC still faces the three major challenges mentioned above. The following path can be considered in future research.

One is that career adaptability is an important variable affecting individual career, and LDC intervention can be designed around career adaptability, such as increasing intervention time; the other is that similar groups may have common characteristics, so future researches can focus on the common needs of clients and carry out more group-based LDC (Maree and Symington, 2015; DaSilva et al., 2020). In addition, a more personalized LDC can be developed by creatively combining counseling methods such as career collages and career portfolios (Barclay, 2019).

Second, it may be a way to cultivate LDC counselors based on degree course and establish the ability framework of professional counselors, due to the general master's or doctor's degree of the LDC counselor in the existing research (Cardoso et al., 2014). In addition, different countries may be able to strengthen the training of LDC counselors through international cooperation, the use of information technology, online and offline training, etc. (Pouyaud et al., 2016; Reid et al., 2016).

Third, in view of the need for evaluation of LDC process and results, more consideration can be given to the comprehensive evaluation. In addition to the existing CCIO and other methods, researchers can learn methods from psychotherapy (Hartung and Vess, 2016; Hartung and Santilli, 2018). Secondly, the use of big data and other forms may also become an important way in the future.

## Author Contributions

YW put forward the core point of this research and wrote the paper. HC, KL, and XG participated in writing and revising the article. All authors listed have made a substantial, direct and intellectual contribution to the work, and approved it for publication.

## Conflict of Interest

The authors declare that the research was conducted in the absence of any commercial or financial relationships that could be construed as a potential conflict of interest.

## References

[B1] Barclay S. R. (2019). Creative use of the career construction interview. Career Dev. Q. 67, 126–138. 10.1002/cdq.12176

[B2] Barclay S. R. Stoltz K. B. (2016a). The life-design group: a case study assessment. Career Dev. Q. 64, 83–96. 10.1002/cdq.12043

[B3] Barclay S. R. Stoltz K. B. (2016b). The life design group: a case study vignette in group career construction counseling. J. Stud. Affairs Res. Pract. 53, 78–89. 10.1080/19496591.2016.1087859

[B4] Barclay S. R. Stoltz K. B. Clemente A. (2019). Assessing manifest interests within the career construction interview. Int. J. Educ. Vocat. Guid. 19, 455–473. 10.1007/s10775-019-09388-1

[B5] Barclay S. R. Wolff L. A. (2012). Exploring the career construction interview for vocational personality assessment. J. Vocat. Behav. 81, 370–377. 10.1016/j.jvb.2012.09.004

[B6] Bikos L. H. Dykhouse E. C. Boutin S. K. Gowen M. J. Rodney H. E. (2013). Practice and research in career counseling and development-2012. Career Dev. Q. 61, 290–329. 10.1002/j.2161-0045.2013.00058.x

[B7] Briddick W. C. Sensoy-Briddick H. Savickas S. (2018). Career construction materials: the story of a career development curriculum in a Turkish school. Early Child Dev. Care 188, 478–489. 10.1080/03004430.2017.142483

[B8] Cardoso P. Duarte M. E. Gaspar R. Bernardo F. Janeiro I. N. Santos G. (2016b). Life design counseling: a study on client's operations for meaning construction. J. Vocat. Behav. 97, 13–21. 10.1016/j.jvb.2016.07.007

[B9] Cardoso P. Gonçalves M. M. Duarte M. E. Silva J. R. Alves D. (2016a). Life design counseling outcome and process: a case study with an adolescent. J. Vocat. Behav. 93, 58–66. 10.1016/j.jvb.2016.01.002

[B10] Cardoso P. Janeiro I. N. Duarte M. E. (2017). Life design counseling group intervention with Portuguese adolescents: a process and outcome study. J. Career Dev. 45, 183–196. 10.1177/0894845316687668

[B11] Cardoso P. Sales C. (2019). Individualized career counseling outcome assessment: a case study using the personal questionnaire. Career Dev. Q. 67, 21–32. 10.1002/cdq.12160

[B12] Cardoso P. Savickas M. L. Goncalves M. M. (2019). Innovative moments in career construction counseling: proposal for an integrative model. Career Dev. Q. 67, 188–204. 10.1002/cdq.12190

[B13] Cardoso P. Savickas M. L. Goncalves M. M. (2020). Facilitating narrative change in career construction counseling. J. Career Dev. 10.1177/0894845319898872. [Epub ahead of print].

[B14] Cardoso P. Silva J. R. Gonçalves M. M. Duarte M. E. (2014). Narrative innovation in life design counseling: the case of Ryan. J. Vocat. Behav. 85, 276–286. 10.1016/j.jvb.2014.08.001

[B15] DaSilva C. S. C. Teixeira M. A. P. Cardoso P. Fernandez-Navarro P. Goncalves M. M. Duarte M. E. (2020). Innovative moments and narrative change in career counselling: a case study. Int. J. Educ. Vocat. Guid. 10.1007/s10775-020-09422-7. [Epub ahead of print].

[B16] Di Fabio A. (2010). Life designing in 21st Century: using a new, strengthened career genogram. J. Psychol. 20, 381–384. 10.1080/14330237.2010.10820389

[B17] Di Fabio A. (2016). Life design and career counseling innovative outcomes. Career Dev. Q. 64, 35–48. 10.1002/cdq.12039

[B18] Di Fabio A. Maree J. G. Goncalves M. M. (2012). Group-based life design counseling in an Italian context. J. Vocat. Behav. 80, 100–107. 10.1016/j.jvb.2011.06.001

[B19] Guichard J. (2009). Self-constructing. J. Vocat. Behav. 75, 251–258. 10.1002/cdq.12040

[B20] Guichard J. (2016a). Reflexivity in life design interventions: comments on life and career design dialogues. J. Vocat. Behav. 97, 78–83. 10.1016/j.jvb.2016.08.001

[B21] Guichard J. (2016b). Life-designing counseling: a comparison of the career construction and self-construction approaches. Psychol. Française, 61, 15–29. 10.1016/j.psfr.2013.03.002

[B22] Guichard J. Pouyaud J. Calan C. D. Dumora B. (2012). Identity construction and career development interventions with emerging adults. J. Vocat. Behav. 81, 52–58. 10.1016/j.jvb.2012.04.004

[B23] Hartung P. J. Santilli S. (2018). My career story: description and initial validity evidence. J. Career Assess. 26, 308–321. 10.1177/1069072717692980

[B24] Hartung P. J. Vess L. (2016). Critical moments in career construction counseling. J. Vocat. Behav. 97, 31–39. 10.1016/j.jvb.2016.07.014

[B25] Jacquin P. Juhel J. (2017). An individual mixed-evaluation method for career intervention. Career Dev. Q. 65, 16–28. 10.1002/cdq.12077

[B26] Jiang Z. Newman A. Le H. Presbitero A. Zheng C. (2019). Career exploration: a review and future research agenda. J. Vocat. Behav. 110, 338–356. 10.1016/j.jvb.2018.08.008

[B27] Lengelle R. Meijers F. Hughes D. (2016). Creative writing for life design: reflexivity, metaphor and change processes through narrative. J. Vocat. Behav. 97, 60–67. 10.1016/j.jvb.2016.07.012

[B28] Maree J. G. (2014). Career construction with a gay client: a case study. Br. J. Guid. Counsel. 42, 436–449. 10.1080/03069885.2014.886670

[B29] Maree J. G. (2015a). Career construction counseling: a thematic analysis of outcomes for four clients. J. Vocat. Behav. 86, 1–9. 10.1016/j.jvb.2014.10.001

[B30] Maree J. G. (2015b). Research on life design in (South) Africa: a qualitative analysis. South Afr. J. Psychol. 2015, 1–17. 10.1177/0081246314566785

[B31] Maree J. G. (2016a). Career construction counseling with a mid-career Black man. Career Dev. Q. 64, 20–34. 10.1002/cdq.12038

[B32] Maree J. G. (2016b). How career construction counseling promotes reflection and reflexivity: two case studies. J. Vocat. Behav. 97, 22–30. 10.1016/j.jvb.2016.07.009

[B33] Maree J. G. (2019). Group career construction counseling: a mixed-methods intervention study with high school students. Career Dev. Q. 67,47–61. 10.1002/cdq.12162

[B34] Maree J. G. (2020a). Innovative career construction counselling for a creative adolescent. Br. J. Guid. Counsel. 48, 98–113. 10.1080/03069885.2018.1504202

[B35] Maree J. G. (2020b). Integrative career counselling for early career individuals. South Afr. J. Psychol. 10.1177/0081246319899592. [Epub ahead of print].

[B36] Maree J. G. Cook A. V. Fletcher L. (2018b). Assessment of the value of group-based counselling for career construction. Int. J. Adolesc. Youth 23,118–132. 10.1080/02673843.2017.1309324

[B37] Maree J. G. Pienaar M. Fletcher L. (2018a). Enhancing the sense of self of peer supporters using life design-related counselling. South Afr. J. Psychol. 48, 420–443. 10.1177/0081246317742246

[B38] Maree J. G. Symington C. (2015). Life design counselling effects on the career adaptability of learners in a selective independent school setting. J. Psychol. Afr. 25, 262–269. 10.1080/14330237.2015.1065076

[B39] Maree J. G. Taylor N. (2016). Development of the maree career matrix: a new interest inventory. South Afr. J. Psychol. 46, 462–476. 10.1177/0081246316641558

[B40] Maree J. G. Twigge A. (2016). Career and self-construction of emerging adults: the value of life designing. Front. Psychol. 6:2041. 10.3389/fpsyg.2015.02041 26793152 PMC4707554

[B41] Millar C. C. Groth O. Mahon J. F. (2018). Management Innovation in a VUCA World: challenges and recommendations. Calif. Manage. Rev. 61, 5–14. 10.1177/00081256188051

[B42] Nota L. Santilli S. Soresi S. (2016). A life-design-based online career intervention for early adolescents: description and initial analysis. Career Dev. Q. 64, 4–19. 10.1002/cdq.12037

[B43] Pouyaud J. Bangali M. Cohen-Scali V. Robinet M. L. Guichard J. (2016). Exploring changes during life and career design dialogues. J. Vocat. Behav. 97, 3–12. 10.1016/j.jvb.2016.07.008

[B44] Reid H. Bimrose J. Brown A. (2016). Prompting reflection and learning in career construction counseling. J. Vocat. Behav. 97, 51–59. 10.1016/j.jvb.2016.07.013

[B45] Rudolph C. W. Lavigne K. N. Zacher H. (2017). Career adaptability: a meta-analysis of relationships with measures of adaptivity, adapting responses, and adaptation results. J. Vocat. Behav. 98, 17–34. 10.1016/j.jvb.2016.09.002

[B46] Savickas M. L. (2008). David V. Tiedeman: engineer of career construction. Career Dev. Q. 56, 217–224. 10.1002/j.2161-0045.2008.tb00035.x

[B47] Savickas M. L. (2011a). constructing careers: actor, agent, and author. J. Employment Counsel. 48, 179–181. 10.1002/j.2161-1920.2011.tb01109.x

[B48] Savickas M. L. (2011b). New questions for vocational psychology: premises, paradigms, and practices. J. Career Assess. 19, 251–258. 10.1177/1069072710395532

[B49] Savickas M. L. (2012). Life design: a paradigm for career intervention in the 21st century. J. Counsel. Dev. 90, 13–19. 10.1111/j.1556-6676.2012.00002.x

[B50] Savickas M. L. (2015). Life-Design Counseling Manual. Retrieved from: http://www.vocopher.com.

[B51] Savickas M. L. (2016). Reflection and reflexivity during life-design interventions: comments on career construction counseling. J. Vocat. Behav. 97, 84–89. 10.1016/j.jvb.2016.09.001

[B52] Savickas M. L. Guichard J. (2016). Symposium introduction: reflexivity in life designing interventions. J. Vocat. Behav. 97, 1–2. 10.1016/j.jvb.2016.07.015

[B53] Savickas M. L. Nota L. Rossier J. Dauwalderr J. Duarte M. E. Guichard J. (2009). Life designing: a paradigm for career construction in the 21st century. J. Vocat. Behav. 75, 251–258. 10.1016/j.jvb.2009.03.004

[B54] Savickas M. L. Porfeli E. J. (2012). Career adapt-abilities scale: construction, reliability, and measurement equivalence across 13 countries. J. Vocat. Behav. 80, 661–673. 10.1016/j.jvb.2012.01.011

[B55] Savickas M. L. Porfeli E. J. Hilton T. L. Savickas S. (2018). The student career construction inventory. J. Vocat. Behav. 106,138–152. 10.1016/j.jvb.2018.01.00923402052

[B56] Savickas M. L. Pouyaud J. (2016). Life design: a general model for career intervention in the 21st century. Psychol. Française 61, 5–14. 10.1016/j.psfr.2013.11.003

[B57] Setlhare M. R Wood L. (2016). Using life design with vulnerable youth. Career Dev. Q. 64, 60–74. 10.1002/cdq.12041

[B58] Taylor J. M. Savickas S. (2016). Narrative career counseling: my career story and pictorial narratives. J. Vocat. Behav. 97, 68–77. 10.1016/j.jvb.2016.07.010

[B59] Tian X. Hou Z. Wang D. Savickas S. Chang X. Cao Y. (2020). Counselor actions to facilitate client change during life-design counseling. Career Dev. Q. 68,48–62. 10.1002/cdq.12212

